# The association between quality measures of medical university press releases and their corresponding news stories—Important information missing

**DOI:** 10.1371/journal.pone.0217295

**Published:** 2019-06-12

**Authors:** Maike Winters, Anna Larsson, Jan Kowalski, Carl Johan Sundberg

**Affiliations:** 1 Department of Public Health Sciences, Karolinska Institutet, Stockholm, Sweden; 2 Department of Clinical Science, Karolinska Institutet, Stockholm, Sweden; 3 National Swedish Broadcasting Corporation, Stockholm, Sweden; 4 JK Biostatistics AB, Stockholm, Sweden; 5 Department of Learning Informatics Management and Ethics, Karolinska Institutet, Stockholm, Sweden; 6 Department of Physiology and Pharmacology, Karolinska Institutet, Stockholm, Sweden; KU Leuven, BELGIUM

## Abstract

**Background:**

The news media is a key source for health and medical information, and relies to a large degree on material from press releases (PR). Medical universities are key players in the dissemination of PRs. This study aims to 1) explore the relation between the quality of press releases (PRs) from medical universities and their corresponding news stories (NSs) and 2) to identify the likelihood that specific scientific and interest-raising measures appear or are omitted in PRs and NSs.

**Methods and findings:**

In this retrospective study using quantitative content analysis, PRs (n = 507) from 21 medical universities in Germany, the Netherlands, Sweden, the USA and the UK were retrieved. Of all PRs, 33% had media coverage, resulting in 496 NSs. With two codebooks, 18 scientific (*e*.*g*. reporting the study design of the study correctly) and 7 interest-raising measures (*e*.*g*. words like ‘ground-breaking’) were evaluated in the PRs and NSs. For all measures the percentage of presence in NSs and PRs was calculated, together with a Mean PR Influence Factor. Quality of PRs and NSs was defined as a score, based on 12 of the 18 scientific measures. Mean (SD) NS quality score was 6.5 (1.7) which was significantly lower than the PR score of 8.0 (1.5). The two quality scores were significantly correlated. Quality measures that were frequently omitted included reporting important study limitations (present in 21% of PRs, 21% of NSs), funding (59% of PRs, 7% of NSs) and conflicts of interest (16% of PRs, 3% of NSs). We did not evaluate the quality of the scientific papers (SPs), and can therefore not determine if the quality of PRs and NSs is associated with the quality of SPs.

**Conclusions:**

This large study of medical university press releases and corresponding news stories showed that important measures of a scientific study such as funding and study limitations were omitted to a very large extent. The lay public and health personnel as well as policy makers, politicians and other decision makers may be misled by incomplete and partly inaccurate representations of scientific studies which could negatively affect important health-related behaviours and decisions.

## Background

The media are an important source of information about developments in science for the general public[1]. Of all scientific disciplines, Europeans reportedly find health and medical care most important[1]. News media have the power not only to influence people’s beliefs and attitudes around medical topics, but also to affect healthcare seeking behaviour[2–6]. For instance, a Danish study found that negative news coverage surrounding statin-use was associated with decreased use of statins, increased myocardial infarction and death from cardiovascular disease[5]. Furthermore, a 10-year analysis of trends of SSRI prescription drug use in the Netherlands and the UK found that changes in its use were associated with a combination of media attention and regulatory warnings[4]. Generally, it has been stated that the more a disease is discussed in the media, the more serious the disease is perceived to be by the lay public[7].

The downside of the media’s influence is apparent when incorrect information is disseminated; once it is widespread, it is very difficult to counter the misinformation[8–10]. A striking example is the reporting about a (fraudulent) study that linked the measles vaccination to autism, which in turn led to anti-vaccination movements and a decrease in vaccine coverage[11]. However, the problem is not restricted to this example. As Sumner et al[12] points out, “the cumulative effects of everyday misreporting can confuse and erode public trust in science and medicine, with detrimental consequences”.

Reporting is assumed to be influenced by the changing media landscape and the working conditions of journalists. Over the past decades, the media landscape has undergone drastic changes. While circulation has shifted from print media to online news, competition has increased and revenues have gone down[13]. Consequent budget cuts have led to layoffs and a higher workload for remaining editorial staff[14]. Journalists’ reliance on public relations material is extensive, as shown in a two-week study of British news[15]. Medical reporting may be especially influenced by such materials with 37% of health news stories (NSs) being based mainly on public relations material[15]. A study examining the media coverage around the association between pancreatic cancer and processed meat, found that only 14% of medical news stories contained a significant amount of original journalism[16]. Press releases (PRs) have thus become an established link between the news media and outside scientific actors, and the information they contain is not unlikely to end up in news stories (NSs)[12].

Given the increased reliance of the media on PRs, it is essential that PRs accurately reflect the scientific papers (SPs) on which they are based. It has been shown that PRs of weak quality are related to the accuracy of their subsequent NSs[17]. In their analysis of PRs from medical universities in the UK, Sumner et al[12] observed that exaggerations in NSs are strongly associated with exaggerations in PRs. In a follow-up study examining PRs from science and medical journals[18], it was confirmed that the PRs appeared to be the source of exaggeration[12,18]. Studies to date have tended to address a few study measures (*e*.*g*. exaggerations or caveats) and have been limited to mainly English-speaking countries.

Accordingly, the present study was broadened to include medical universities from five countries with four languages and was extended to include a larger set of quality measures, with the aims to 1. explore the relation between quality measures of press releases and their corresponding news stories and 2. identify the likelihood that specific scientific and interest-raising measures appear or are omitted in PRs and NSs.

## Methods

Press releases (PRs) were retrieved from the 1^st^ of March 2015 until the 30^th^ of June 2015, from the websites of four high-ranked universities with medical faculties (according to the Times World University ranking of 2014)[19] from Germany, the Netherlands, Sweden, the USA and UK. Because the number of PRs from one of the selected universities in the Netherlands was so low (n = 1), one extra Dutch university was included. The press releases were included in the study if the topic was medical and directly related to a published scientific paper (SP) in a recognized scientific journal, yielding a total of 507 PRs (see flowchart in S1 Fig). Media coverage was captured by searching the media databases LexisNexis and Retriever, as well as by searching through Google News. Tag words or words from the PR headline were used with date limits from three days before the release date of the PR up till two weeks after. By using these databases, news stories (NSs) published in print and/or online could be included. For the US and the UK, only English-language media were selected. For Germany, the Netherlands and Sweden, English-language media was searched as well as media in the main official language of the country (*i*.*e*. German, Dutch and Swedish respectively). NSs were included if the published SP was discussed. Opinion pieces and pieces written by the authors of the SP were excluded. One PR can have a varying number of related NSs. When news goes viral, it is not uncommon that media outlets use other media and press agencies as sources[16]. Because it was beyond the aim and scope of this study to analyse the viral flow of media, a maximum was set of 12 NSs per PRs. If there were more than 12 NSs for one PR, priority was given to the highest ranked articles in the used databases. Media outlets can reprint material from press agencies; different outlets could therefore print the same NSs. If this was the case, only the original press agency article was included.

### Codebooks

This study assumes a linear flow of information, from SP to PR to NS (see S2 Fig)–much like the transmission model of McQuail[20]. Two codebooks were developed to analyse this flow; the first codebook compared the PR with the SP and the second codebook compared the NS with the PR, see S2 Fig. Codebook development was based on previously published studies[12,17,21] and was modified to include questions regarding the reporting of 18 scientific measures (*e*.*g*. study design, main aim) in the SP, PR and NS. Furthermore, we explored the use of 7 interest-raising measures (*e*.*g*. use of the word ‘first’ or ‘new’ or ‘ground-breaking’ words) in the PR and NS.

The codebooks were pilot tested by two senior researchers. Coders using the second codebook were blinded to the scientific paper, as a way to simulate how journalists would likely interpret the press release. All press releases and news stories were then coded by a team of five coders, who received a one-day training prior to commencing the coding for the study. All coders were proficient English language users and everybody spoke at least one of the other languages of the study fluently (*i*.*e*. Dutch, Swedish, German). There were at least two coders for every language.

### Scientific and interest-raising measures

For all 18 scientific and seven interest-raising measures, it was determined whether they were reported (correctly) in the PR and NS, according to the comparison criteria listed in S1 Table. This enabled a comparison between the proportion of a measure being correctly mentioned in the PR with the proportion in the NS. To determine whether the relationship independent (IV) and dependent variables (DV) were exaggerated, we analysed whether the PR and/or NS made a different claim than the SP (*e*.*g*. an SP states a correlational relationship and the PR writes a statement of ‘can’).

### Quality measures

Of the 18 scientific measures, 12 were deemed essential for a high-quality PR and NS and formed the basis of the quality scores. The 12 quality measures were based on previous literature[12,17,21] and consultation with senior researchers. They included: the correct reporting in the PR and/or NS of: main aim, study design, independent (IV) and dependent variables (DV) (exposure and outcome) and their relationship, sample size, main results, quantification of results, main conclusion, most important limitations, funding and conflict of interest (see S1 Table). Depending on the study design, different statements can be made about the strength of the relationship between the IV and the DV. We have used seven categories, mirroring the approach by Sumner et al[12], namely: no statement about relationship, statement of no relationship, statement of correlation, ambiguous statement of relationship, conditional statement of relationship, statement of ‘can’ and statement of causation.

### Statistics

To explore the inter-rater reliability, 25% of the PRs and NSs were randomly selected for double-coding (*i*.*e*. coded by two coders). They were further analysed using the kappa statistic for inter-rater reliability as well as the percentage agreement in instances where kappa could not be calculated. The overall kappa for the two codebooks was 0.53, percent agreement was higher at 0.85, indicating moderate agreement[22].

The proportion of a scientific measure correctly reported was defined as a quality measure being observed/present and correctly reported in the NS and PR respectively. Further, PRs and NSs received a score ranging from 0 to 12 based on the number of quality measures correctly reported. The comparison between the PR and NS distribution was done using the two-sided Wilcoxon signed-rank test. The correlation between the quality measures of PRs and NSs was estimated using the Spearman rank-order correlation coefficient. The distribution of the number of PRs and NSs was presented in tables and graphically described.

For derivation of the scientific measure regarding the exaggerated relationship between dependent and independent variables, we selected a subgroup of SP that did not report causal statement of relationship between the variables and calculated the same statistics as above. Similarly, the analysis of conflict of interest was derived using a subgroup of SPs that declared a potential conflict of interest.

The presence of a single measure in an NS (observed number/total number) within the corresponding PR was defined as the percentage of presence. The mean percentage of presence was further calculated for the aggregated number of all PRs. The calculation was repeated for all 18 scientific and 7 interest-raising measures. Calculation was done both when the measure was present as well as when absent in PRs. Next, the Mean PR Influence Factor was calculated per measure, dividing the mean percentage of presence in NS when the measure was present in the PR by the mean percentage of presence in NS when the measure was absent in the PR. The difference between mean percentage of presence in NS (PR present / PR absence) was calculated using the two-sided t-test, where p<0.05 was defined as statistically significant.

## Results

A total of 507 Press releases (PRs) were retrieved (see S3 Table), of which 170 (34%) had media coverage, with at least one news story (NS) identified as related to the publication of the PR. These 170 PRs had a total of 496 NSs (S3 Table). The USA had the largest number of PRs (n = 67), as well as the largest number of corresponding NSs from the PRs (n = 203). There were only 6 PRs (12%) from the 4 German universities that had any media coverage. Sweden had the highest relative media coverage of the PRs: 43%. The mean number of NSs per PR was 2.89 (median 2).

### Quality measures

**Fig 1:**
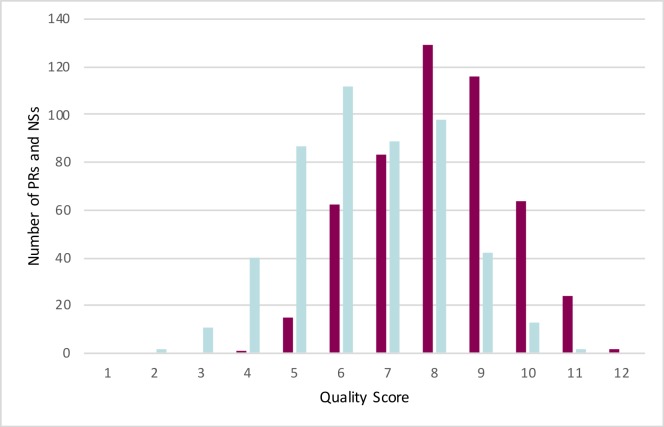
Histogram for the aggregated number of quality measures in PRs & NSs. Purple bars: PRs, blue bars: NSs.

Out of a total of 12 quality measures, PRs scored a mean of 8.0 (SD: 1.5, range: 4–12), whereas the NSs scored a mean of 6.5 (SD: 1.7, range: 2–11). Fig 1 and S4 Table show that the PRs reported significantly more (p<0.001) quality measures than the NSs. Quality measures in PRs and NSs were correlated (Spearman’s Rho 0.35, p<0.001).

Four of the 12 quality measures were reported in more than 90% of the PRs (*i*.*e*. independent variable (IV), dependent variable (DV), main conclusions, main results), see Table 1.

**Table 1:** Proportion of quality measures correctly reported in PRs and NSs.

Measures	Present in PR (n = 170) Number (%)	Present in NS (n = 496) Number (%)
Main aim	125 (74)	158 (32)
Study design	143 (84)	310 (63)
Independent variable (IV)	163 (96)	406 (82)
Dependent variable (DV)	156 (92)	415 (84)
Relationship IV-DV	82 (48)	266 (54)
Sample size	109 (64)	308 (62)
Main results	170 (100)	485 (98)
Quantification of results	102 (60)	273 (55)
Main conclusions	157 (92)	475 (96)
Most important limitations	36 (21)	105 (21)
Funding	100 (59)	33 (7)
Conflict of interest^a^^,^^b^	9 (16)^a^	5 (3)^b^

^a^Number of relevant PRs: 55

^b^Number of relevant NSs: 170

Of those, two were reported in more than 90% of the NSs (main conclusions and main results). Measures that were frequently omitted included mentioning important limitations (present in 21% of both the PRs and NSs) and funding (present in 59% of PRs, 7% of NSs). Furthermore, potential conflict of interest was mentioned in 16% of the PRs and 3% of the NSs, in a sample of 55 SPs that declared a potential conflict of interest.

### Scientific and interest-raising measures

Descriptive statistics of all other scientific and interest-raising measures are summarized in Table 2.

**Table 2:** Proportion of scientific and interest-raising measures correctly reported in PRs and NSs.

**Scientific Measures**	**Reported in PR** **(n = 170)** **No (%)**	**Reported in NS** **(n = 496)** **No (%)**
Relationship IV-DV in title	59 (35)	145 (29)
Control group	110 (65)	180 (36)
Base rate	55 (32)	178 (36)
IV in title	144 (85)	325 (66)
DV in title	143 (84)	291 (59)
Exaggeration IV-DV^a^^,^^b^	24 (34)^a^	132 (60)^b^
**Interest-Raising Measures**	**Reported in PRs** **(n = 170)** **No (%)**	**Reported in NSs** **(n = 496)** **No (%)**
Quotes	168 (99)	447 (90)
Ground-breaking words in text	11 (7)	52 (11)
Ground-breaking words in quotes	14 (8)	37 (8)
Advice in quotes	59 (35)	69 (14)
Use of the word ‘first’	75 (44)	95 (19)
Use of the word ‘new’	130 (77)	273 (55)
Subjective words in text	69 (41)	251 (51)

^a^Number of relevant PRs: 70

^b^Number of relevant NSs: 219

In general, scientific measures such as reporting the control group of the study were more often (p<0.001, Table 3) mentioned in PRs (65%) than in NSs (36%), whereas interest-raising measures such as subjective wording were more frequently (p = 0.03, Table 3) reported in the NSs (51%) than in PRs (41%).

**Table 3:** Mean percentage of presence in NSs and mean PR influence factor per measure.

**Quality Measures** **Measure present in PR**	**Number** **Of PRs**	Mean Percentage of Presence in NSs (%)*	Mean PR Influence Factor**	**P-value**
Main Aim				
Yes	125	42	1.7	0.02
No	45	25		
Study Design				
Yes	143	67	1.5	0.01
No	27	44		
Independent Variable (IV) in Text				
Yes	163	84	1.3	0.14
No	7	65		
Dependent Variable (DV) in Text				
Yes	156	85	0.9	0.38
No	14	92		
Relationship IV-DV in text				
Yes	82	69	1.4	0.001
No	88	48		
Sample Size				
Yes	109	75	2.1	<0.001
No	61	35		
Main Results				
Yes	170	98	-	-
No	0	-		
Quantification of Results				
Yes	102	82	14.1	<0.001
No	68	6		
Main Conclusions				
Yes	157	95	1.2	0.01
No	13	78		
Important Limitations				
Yes	36	48	4.3	<0.001
No	134	11		
Funding				
Yes	100	8.6	1.7	0.30
No	70	5.0		
Conflict of Interest^1^				
Yes	9	15	5.7	0.08
No	46	3		
**Study Measures**	**Number** **Of PRs**	Mean Percentage of Presence in NSs (%)*	Mean PR Influence Factor**	**P-value**
Relationship IV-DV in Title				
Yes	59	42	1.7	0.004
No	111	25		
Exaggeration Relationship IV-DV^2^				
Yes	24	79	1.5	0.03
No	46	52		
Control Group				
Yes	110	53	6.1	<0.001
No	60	9		
Base Rate				
Yes	56	69	3.2	<0.001
No	114	22		
Independent Variable (IV) in Title				
Yes	144	71	1.5	0.003
No	26	46		
Dependent Variable (DV) in Title				
Yes	143	60	1.2	0.24
No	27	50		
**Interest-Raising Measures**	**Number** **Of PRs**	Mean Percentage of Presence in NSs (%)*	Mean PR Influence Factor**	**P-value**
Quotes				
Yes	168	89	1.1	0.79
No	2	84		
Ground-breaking Words in Text				
Yes	11	15	1.6	0.50
No	159	10		
Ground-breaking Words in Quotes				
Yes	14	12	1.6	0.97
No	156	8		
Advice in Quotes				
Yes	59	23	2.9	<0.001
No	111	8		
Use of the Word ‘First’				
Yes	75	31	4.7	<0.001
No	95	7		
Use of the Word ‘New’				
Yes	130	61	1.6	0.01
No	40	38		
Subjective Words in Text				
Yes	69	52	1.4	0.03
No	101	38		

*Mean percentage of presence in NSs, aggregated for all PRs. Calculation was done for PRs where the measure was present as well as PRs where the measure was absent.

**Calculated as the ratio between mean percentage of presence in NS when present in PR and mean percentage of presence in NS when absent in PR

^1^Analysis included on PRs that were based on SPs that declared a potential conflict of interest

^*2*^Analysis included only PRs that were based on SPs that did not mention a causal relationship between IV and DV

**Fig 2:**
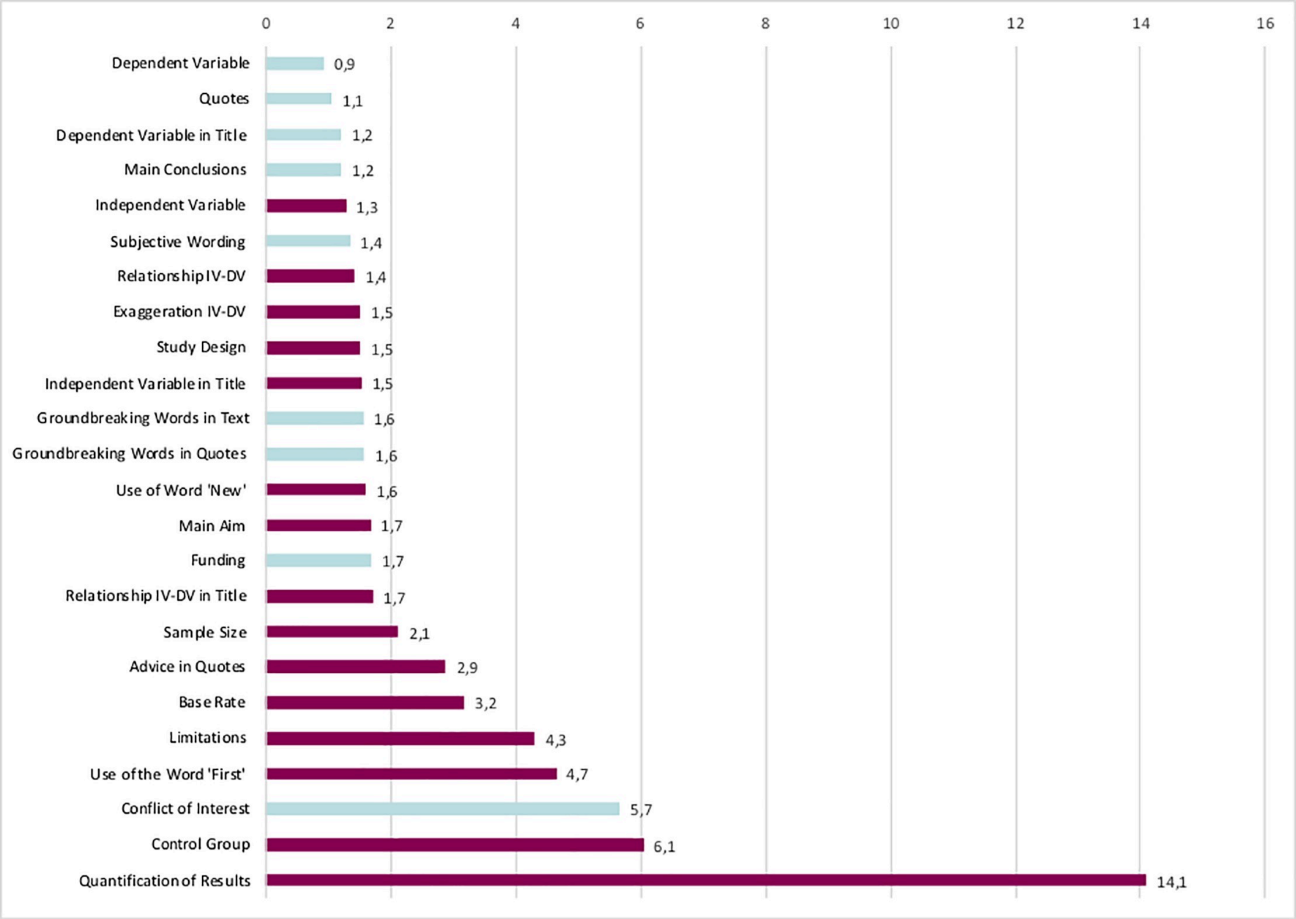
Mean PR influence factor by measure. Blue bars: p>0.05. Purple bars: p<0.05 Mean PR Influence Factor is the ratio between mean percentage of presence in NSs (PR presence / PR absence).

Table 3 summarizes the mean percentage of presence in NSs and the Mean PR Influence Factors, which are graphically presented in Fig 2 and S3 Fig.

The probability of being reported in the NS was higher for all measures when reported in the PR, except for the correct mentioning of the DV (mean percentage of reporting in NSs: 85% when present in PRs, 92% when absent in PRs). The following measures had the highest Mean PR Influence Factors: quantification of the results (Mean PR Influence Factor: 14.1, p<0.001), mentioning the control group of the study (Mean PR Influence Factor: 6.1, p<0.001), mentioning a conflict of interest (Mean PR Influence Factor 5.7, p = 0.08), mentioning the word ‘first’ at least once (Mean PR Influence Factor: 4.7, p<0.001) and mentioning limitations of the study (Mean PR Influence Factor: 4.3, p<0.001).

Exaggeration of the relationship between IV-DV was found in 34% of the PRs and 60% of the NSs (Table 2), analysed in a limited sample of 70 SPs that did not make a causal statement. There was a significantly higher chance of an exaggerated claim in an NS when there was an exaggerated claim in the PR (Mean PR Influence Factor: 1.53, p = 0.03), see Table 3.

## Discussion

This large, five-country study shows that the quality scores of medical university press releases (PRs) and their related news stories (NSs) were significantly correlated and that the quality of NSs was lower than the quality of the PRs. Also, as information about a scientific publication (SP) passes to PRs and subsequent NSs, more quality measures about a scientific study were omitted. In both PRs and NSs, the most commonly omitted quality measures were limitations of the scientific study, sources of funding of the study and conflicts of interest.

Omissions can be partially explained by framing. Through the selective highlighting of certain pieces of information and omitting others, PRs try to portray a positive picture of the SP[23]. Because PRs are part of the universities’ branding strategies, with the aim to build a strong, credible brand[24,25], PRs are especially prone to framing and omissions. In trying to increase the chance of media uptake, PRs are usually written according to journalistic guidelines[26,27]. Potentially negative aspects of an SP, such as limitations, are frequently left out[28] as it is feared that this might undermine the credibility of the study[12]. However, this fear bears no substance as it is found that reporting caveats of a study actually improves the credibility of both scientists and journalists[29] and does not reduce the uptake of the news by journalists[18]. Framing and its consequent omissions of certain measures can mislead an audience. Even though the information presented in the PR is in a literal sense not false, omissions and framing may cause an audience to interpret the message differently[30,31]. Incomplete reporting and misinformation can contribute to distrust in the media and in science[32,33]. This is especially worrisome in the ‘post-truth era’[9], where misinformation is prevalent[10,34].

The Mean PR Influence Factors showed the substantial influence of PRs on NSs on most of the analysed measures. We found that exaggeration of the relationship between IV and DV occurred in 34% of the PRs and that NSs are significantly more likely to take up exaggerations when previously reported in PRs. Previous research also suggest that PRs seem to be the source of exaggerations[12,18,35]. The social amplification of risk framework posits that the public’s perception of risk (or hope) can be amplified or attenuated by social processes–the news media play an important role in this[36]. Overstating the relationship between IV-DV in PRs and NSs, versus the finding in the SPs, can skew the perception of scientific results[36,37], from which fearmongering, or unrealistic hope for new treatments, can follow.

Universities in the USA and the UK had the highest output of PRs, and Germany and the Netherlands had a low number of PRs. The reason for this may be that branding strategies are different. The USA has had a longer tradition of higher education branding[24], which can in part be explained by the financial structures of American universities[38]. It is difficult to explain the relatively low production and uptake of PRs in Germany, the largest and most diverse print media landscape in Europe[39]. It may be speculated that the low uptake of PRs might be related to the finding that 84% of German journalists see their role as ‘providing an analysis of current affairs’, a substantially higher figure than in the other countries in this study[40].

### Practical implications

Considering the importance of media reporting on health literacy and behaviour[3] and the clear association between the quality of NSs and PRs, both press officers and journalists should make more effort to portray results of a scientific study as accurately and completely as possible. Furthermore, as PRs usually cover the publication of one SP, it is advisable to contextualize the findings as much as possible.

### Strengths and limitations

A major strength of this study was the inclusion of PRs and NSs from multiple (five) countries, which provided a broader generalizability than previous studies. To our knowledge, this is the first study that included PRs and NSs in four different languages. Also, the broadened scope with 18 scientific measures and 7 interest-raising measures, enabled a more extensive overview of measures of interest.

Limitations of this study include the moderate level observed in the kappa statistics for agreement, which was lower than the kappas reported in other studies[12,18,35]. This could partly be a reflection of the extensiveness of the codebooks. In practice however, reliability was higher as coders discussed some uncertainties until agreement was reached. Still, coders might interpret study measures differently. A potential bias stemming from this could have diluted the results to some extent.

The flow of information was assumed to be linear: from SP to PR to NS[20]. In reality, this model is more complex. Current affairs influence publication and framing decisions. Furthermore, the PR might not be the only source of inspiration for an NS–it has been shown that publications by other media also play an influential role[16].

Whereas we evaluated the quality measures of PRs and NSs, we did not study the quality of SPs. A recent study shows that exaggerations of the relationship between IV and DV occurred in 34% of a sample of SPs[37], providing evidence that the quality of PRs and NSs could already be influenced by the quality of SPs. Furthermore, a critical assessment of SPs would be justified, given the likelihood of false findings and publication bias[41,42].

## Conclusions

This large multi-national study shows that there was a correlation between the quality of medical university press releases and related news stories. Also, measures were more likely to be reported in NSs if reported in PRs and important measures such as potential conflicts of interest, funding and study limitations were omitted to a very large extent. Altogether, the content and quality of NSs seem to be influenced by how medical university PRs are written and framed. This may have serious repercussions since the lay public, health personnel as well as policy makers, politicians and other decision makers, may be misled by incomplete and partly inaccurate representations of scientific studies which could negatively affect important health-related behaviours and decisions.

## Supporting information

S1 Table Scientific and interest-raising measures evaluated in PRs and NSs. (PDF)

S2 Table Universities and PRs by country. (PDF)

S3 Table News story coverage by country. (PDF)

S4 Table Distribution of the aggregated number of quality measures reported in PRs and NSs. (PDF)

S1 Fig Flowchart of the study. (PDF)

S2 Fig Flow of information and codebooks. (PDF)

S3 Fig Mean percentage of presence of measures in NSs by presence or absence in PRs. (PDF)

S1 File Replication dataset. (SAV)
