# Supplementary material for: The association between quality measures of medical university press releases and their corresponding news stories—Important information missing
Source: PLoS One. 2019 Jun 12;14(6):e0217295. doi: 10.1371/journal.pone.0217295 (PMC6561540; doi:10.1371/journal.pone.0217295)
Supplement: S1 Table — (PDF) [file pone.0217295.s001.pdf]

S1 Table. Scientific and Interest-Raising Measures evaluated in PRs and NSs

Quality Measures	Evaluation in codebooks
Main aim	Correctly mentioned in PR and NS
Study design	Correctly mentioned in PR and NS
Independent variable (IV) in text	Correctly mentioned in PR and NS
Dependent variable (DV) in text	Correctly mentioned in PR and NS
Relationship IV/DV in text	Correctly mentioned the relationship between IV and DV in text of PR (compared with SP) and NS (compared with PR)
Sample size	Correctly mentioned in PR and NS
Main results	Correctly mentioned in PR and NS
Quantification of results	Correctly mentioned in PR and NS
Main conclusions	Correctly mentioned in PR and NS
Most important limitations	Correctly mentioned in PR and NS
Funding	Correctly mentioned in PR and NS
Conflict of interest	Correctly mentioned in PR and NS, sample restricted to include only SPs that declared any conflict of interest

Scientific Measures	Evaluation in codebooks
Relationship IV/DV in title	Correctly mentioned the relationship between IV and DV in title of PR (compared to SP) and NS (compared to PR)
Exaggeration IV/DV	Stronger statement of relationship between IV and DV in PR (as compared to SP) and NS (as compared to PR): only SPs included that did not contain a causal statement
Control group	Correctly mentioned in PR and NS
Base rate	Correctly mentioned in PR and NS, defined as a prevalence or incidence of a disease/condition
IV in title	Correctly mentioned in the title of PR and NS
DV in title	Correctly mentioned in title of PR and NS

Interest-Raising Measures	Evaluation in codebooks
Quotes	Presence of quotes in PR and NS
Groundbreaking words in text	Use of at least 1 word such as 'groundbreaking, 'game-changer' etc. in the text PR and NS
Groundbreaking words in quotes	Use of at least 1 word such as 'groundbreaking', 'game-changer' etc. in the quotes PR and NS
Advice in quotes	Advice being given in the quotes in PR and NS
Use of the word 'first'	Mentioning at least once the word 'first' in PR and NS
Use of the word 'new'	Mentioning at least once the word 'new' in PR and NS
Subjective words in text	Mentioning of subjective words such as 'great' at least 1 time in text of PR and NS
