# Supplementary material for: The association between quality measures of medical university press releases and their corresponding news stories—Important information missing
Source: PLoS One. 2019 Jun 12;14(6):e0217295. doi: 10.1371/journal.pone.0217295 (PMC6561540; doi:10.1371/journal.pone.0217295)
Supplement: S2 Table — (PDF) [file pone.0217295.s002.pdf]

S2 Table. Universities and PRs by country

Country	University	Number of PRs
Germany	Heidelberg University	11
	Ludwig Maximilian University of Munich	17
	Technical University of Munich	8
	Charité Berlin (Humboldt University & Freie University)	14
Netherlands	Leiden University	19
	Utrecht University	5
	University of Amsterdam	4
	Erasmus University Rotterdam	1
	Maastricht University	7
Sweden	Karolinska Institutet	20
	Lund University	20
	Uppsala University	13
	University of Gothenburg / Sahlgrenska	27
UK	University of Oxford	19
	University of Cambridge	39
	University College London	29
	Imperial College London	36
USA	Harvard University	63
	Stanford University	35
	University of California, Los Angeles	47
	Johns Hopkins University	74
