# Supplementary material for: The association between quality measures of medical university press releases and their corresponding news stories—Important information missing
Source: PLoS One. 2019 Jun 12;14(6):e0217295. doi: 10.1371/journal.pone.0217295 (PMC6561540; doi:10.1371/journal.pone.0217295)
Supplement: S3 Table — (PDF) [file pone.0217295.s003.pdf]

S3 Table. News Story coverage by country

Country	Total Number of PRs	PRs with at least 1 corresponding NS No (%)	Number of NSs from PR
Germany	50	6 (12)	12
Netherlands	36	13 (36)	42
Sweden	80	34 (43)	84
UK	123	50 (41)	155
USA	219	67 (31)	203
**Total**	**508**	**170 (33)**	**496**
