# Supplementary material for: The association between quality measures of medical university press releases and their corresponding news stories—Important information missing
Source: PLoS One. 2019 Jun 12;14(6):e0217295. doi: 10.1371/journal.pone.0217295 (PMC6561540; doi:10.1371/journal.pone.0217295)
Supplement: S4 Table — (PDF) [file pone.0217295.s004.pdf]

S4 Table. Distribution of the aggregated number of quality measures reported in PRs and NSs

Quality Measures	Number of Press Releases	Number of News Stories	Cumulative Number of PRs	Cumulative Number of NSs
0	0	0	0	0
1	0	0	0	0
2	0	2	0	2
3	0	11	0	13
4	1	40	1	53
5	15	87	16	140
6	62	112	78	252
7	83	89	161	341
8	129	98	290	439
9	116	42	406	481
10	64	13	470	494
11	24	2	494	496
12	2	0	496	496
