# Supplementary figures and images for: The association between quality measures of medical university press releases and their corresponding news stories—Important information missing
Source: PLoS One. 2019 Jun 12;14(6):e0217295. doi: 10.1371/journal.pone.0217295 (PMC6561540; doi:10.1371/journal.pone.0217295)

**S1 Fig. Flowchart of the study**

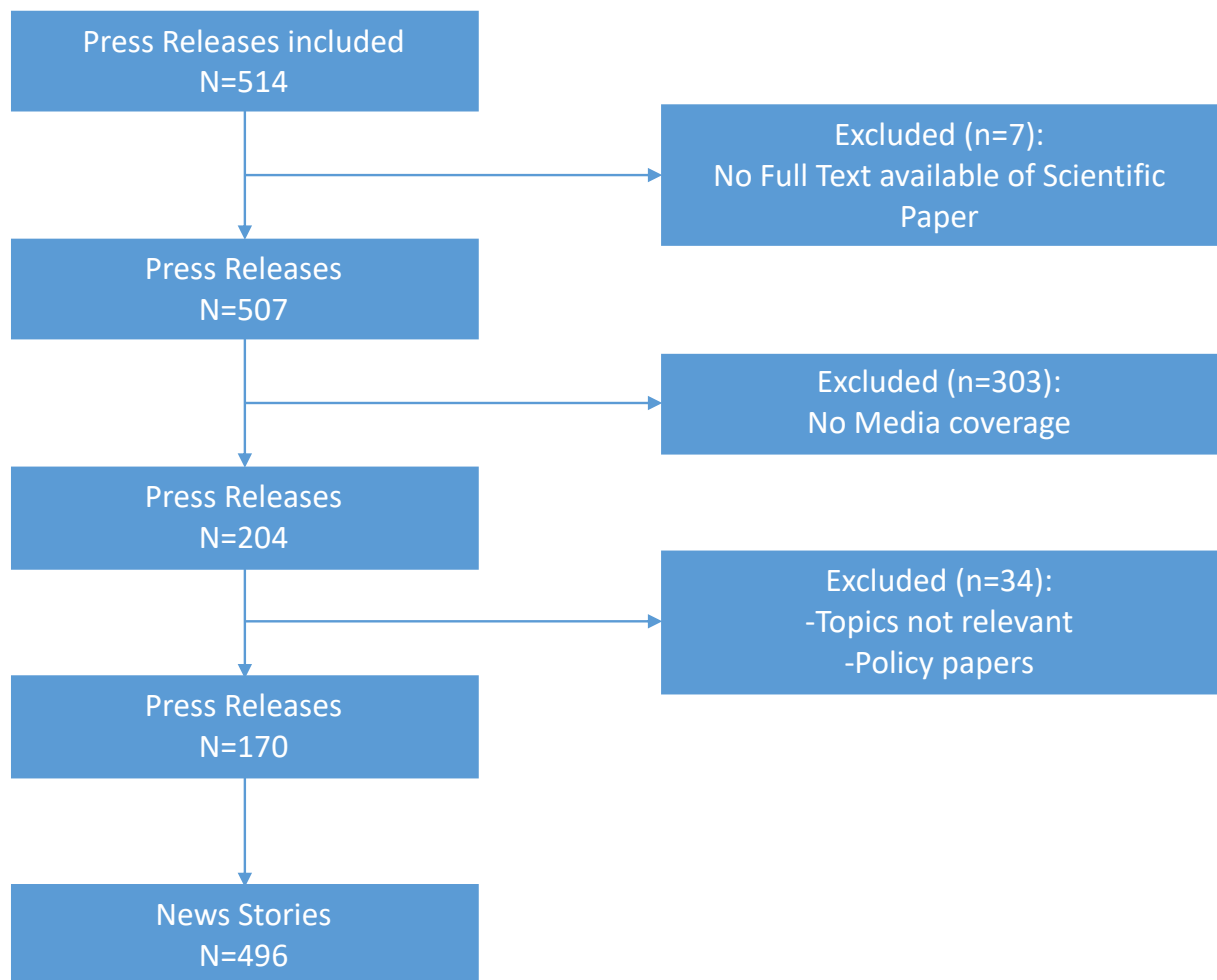

Supplement: S1 Fig — (PDF) [file pone.0217295.s005.pdf]

**S2 Fig. Flow of information and codebooks**

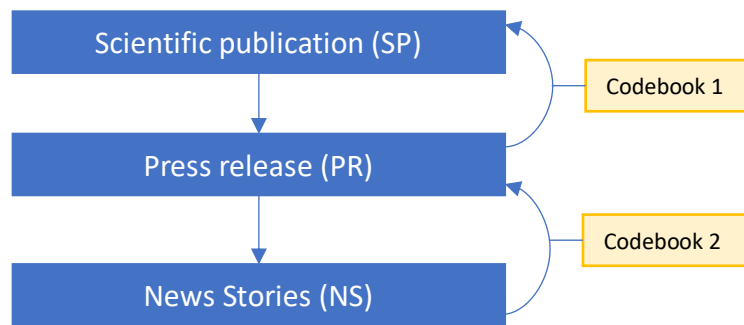

Supplement: S2 Fig — (PDF) [file pone.0217295.s006.pdf]

**S3 Fig. Mean Percentage of Presence of Measure in NSs by Presence or Absence in PRs**

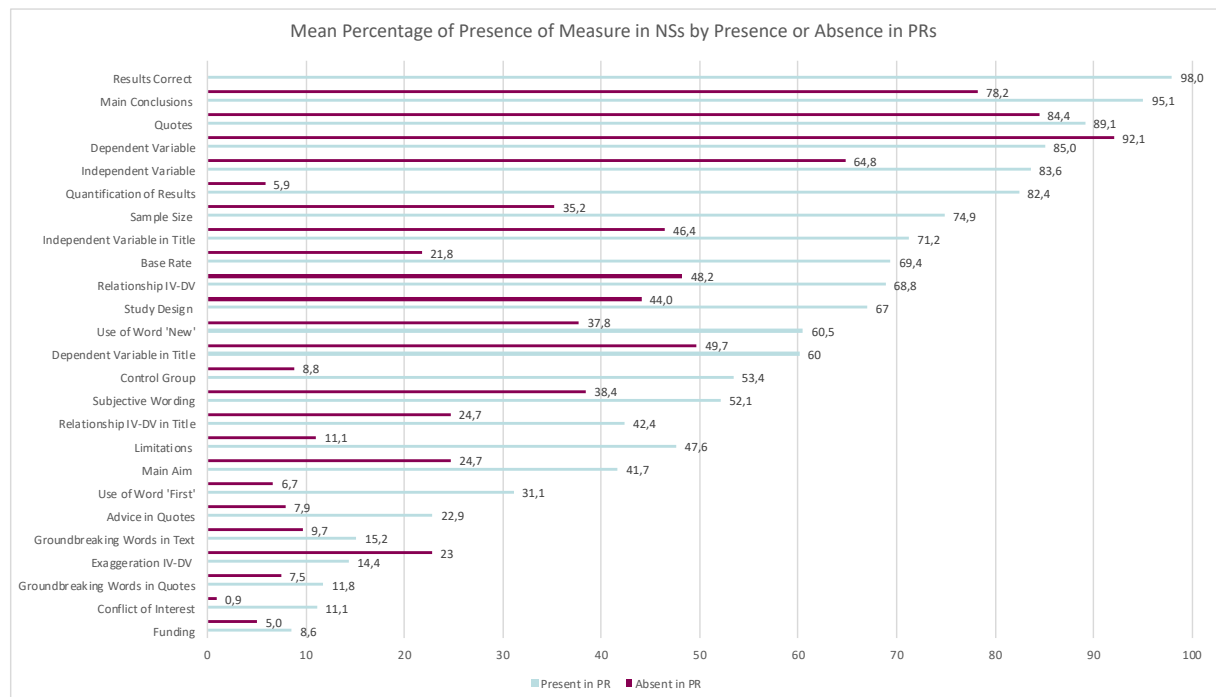

Supplement: S3 Fig — (PDF) [file pone.0217295.s007.pdf]
